# Effect of lifestyle intervention on the risk of incident diabetes in individuals with impaired fasting glucose and low or high genetic risk for the development of type 2 diabetes in men: a T2D-GENE trial

**DOI:** 10.29219/fnr.v65.7721

**Published:** 2021-09-23

**Authors:** Ursula Schwab, Maria Lankinen, Markku Laakso

**Affiliations:** 1 School of Medicine, Institute of Public Health and Clinical Nutrition, University of Eastern Finland, Kuopio, Finland; 2 Department of Medicine, Endocrinology and Clinical Nutrition, Kuopio University Hospital, Kuopio, Finland; 3 Institute of Clinical Medicine, Internal Medicine, University of Eastern Finland, Kuopio Campus and Kuopio University Hospital, Kuopio, Finland

**Keywords:** diet, genotype, genetic risk, glucose metabolism, physical activity, type 2 diabetes

## Abstract

**Purpose:**

Genetic and lifestyle/environmental factors as well as their interplay contribute to the pathogenesis of type 2 diabetes (T2D). Several trials have shown that lifestyle intervention is effective in the prevention of T2D, but there are no trials that have taken into account the genetic risk of the participants. The aim of our T2D-GENE trial (ClinicalTrials.gov ID: NCT02709057) is to investigate the effects of lifestyle intervention on the prevention of T2D in participants with a high genetic risk of T2D compared with participants with a low genetic risk of T2D.

**Methods:**

Both intervention and control groups include 300 participants with low and 300 participants with high genetic risk for T2D. Genetic risk was evaluated by genetic risk score, and these two groups were matched additionally for fasting plasma glucose concentration, age, and body mass index. Corresponding control groups (300 participants each) do not have lifestyle intervention. The inclusion criteria are impaired fasting glucose at entry with or without impaired glucose tolerance, age 50–75 years, and body mass index ≥25 kg/m^2^. The primary outcome is incident T2D and the intervention lasts for 3 years.

**Conclusion:**

If the effects of the lifestyle intervention are independent from the genetic risk of the participants, our study will be of great importance for the entire T2D research community, health care providers, and individuals at high risk for T2D. In this case, lifestyle intervention is beneficial for all individuals at risk for developing T2D, independently of genetic risk.

**ClinicalTrials.gov ID:**

NCT02709057 March 15, 2016

The prevalence of diabetes mellitus will likely increase globally from 463 million individuals in 2019 to 700 million individuals in 2045 (1). This epidemic is mainly attributable to type 2 diabetes mellitus (T2D), which represents about 90–95% of all cases. T2D is a metabolic disorder characterized by chronic hyperglycemia and defects in insulin secretion, insulin action, or both. Genetic and lifestyle/environmental factors as well as their interplay contribute to the pathogenesis of T2D. Genome-wide association studies have identified so far >400 variants for T2D (2, 3). Almost all genetic variants regulate insulin secretion, and only a few of them regulate insulin sensitivity.

Several studies have indicated that lifestyle intervention is effective in the prevention of T2D. The first two of these randomized intervention studies, the Finnish Diabetes Prevention Study (Finnish DPS) and the Diabetes Prevention Program (DPP) from the USA (4, 5), showed that lifestyle intervention lowered the risk of T2D by 58% compared with the control group. In the Finnish DPS study (4), only people with impaired glucose tolerance (IGT) were included, and in the DPP study, people with both impaired fasting glucose (IFG) and IGT were accepted as entry criteria (5). Isolated IFG is a prediabetic condition where only fasting glucose is elevated caused by increased gluconeogenesis. Additionally, beta cell function is impaired, and glucagon secretion is inappropriately elevated (6). In contrast, people with combined IFG and IGT have a combination of increased gluconeogenesis, lack of suppression of glycogenolysis by insulin, impaired glucose disposal with extrahepatic insulin resistance, and impaired insulin secretion (7).

Several diabetes prevention trials have been published on the effects of lifestyle changes (diet and physical activity), and a meta-analysis of these trials reported a 57% reduction in incident T2D (8). There are no previous trials investigating the effect of the genetic background of the participants (the number of the risk alleles for T2D) on the effect of the prevention of T2D. Therefore, the aim of the T2D-GENE trial is to investigate the effects of lifestyle intervention (diet, physical activity, and weight maintenance/weight loss) on the prevention of incident T2D and the worsening of hyperglycemia in people with a high number of T2D risk alleles compared with people with a low number of T2D risk alleles. The second aim of our study is to investigate whether a group-based intervention utilizing a website is as effective as is an individual-based intervention. The hypotheses of the study are that lifestyle intervention is efficient independent of the number of T2D risk alleles (high and low genetic risks of T2D), and that a group-based intervention is as effective as an individual-based intervention in the prevention of T2D.

The primary outcome of our study is incident T2D defined by fasting plasma glucose ≥7.0 mmol/l or 2-h glucose ≥11.1 mmol/l in an oral glucose tolerance test (OGTT) or HbA1c ≥ 47 mmol/mol (6.5%), according to the American Diabetes Association criteria (9), or drug treatment for T2D started during a 3-year intervention trial. The secondary outcomes are changes in glucose area under the curve (AUC) in an OGTT, changes in insulin secretion and insulin sensitivity, and incident cardiovascular disease (CVD) events (fatal or non-fatal myocardial infarction, fatal or non-fatal stroke, death from cardiovascular causes or incident heart failure).

## Participants and methods

### Participants

Study participants have been selected from the Metabolic Syndrome in Men (METSIM) study, which includes 10,197 men, aged from 45 to 73 years at baseline, randomly selected from the population register of the Kuopio town (baseline examination in 2005–2010) (10). The aim of the METSIM study is to investigate genetic and non-genetic factors associated with the risk of T2D, CVD, and insulin resistance-related traits.

The *inclusion criteria* for the present study were 1) IFG at entry (fasting plasma glucose 5.6–6.9 mmol/l) and with (2-h glucose 7.8–11.0 mmol/l) or without IGT (2-h glucose <7.8 mmol/l), and HbA1c <48 mmol/mol (<6.5%), 2) age 50–75 years, 3) body mass index (BMI) ≥25 kg/m^2^, and 4) the genetic risk score (GRS) (low risk 1–2 quintiles of the GRS and high risk 4–5 quintiles of the GRS). The *exclusion criteria* were 1) age <50 years or >75 years, 2) BMI <25 kg/m^2^, 3) type 1 or type 2 diabetes or isolated IGT (2-h plasma glucose <5.6 mmol/l and 2-h plasma glucose 7.8–11.0 mmol/l) or HbA1c ≥48 mmol/mol (≥6.5%), and 4) chronic diseases preventing to participate in the trial. After the identification of potential participants of the METSIM study, they were approached by an invitation letter.

**Fig. 1:**
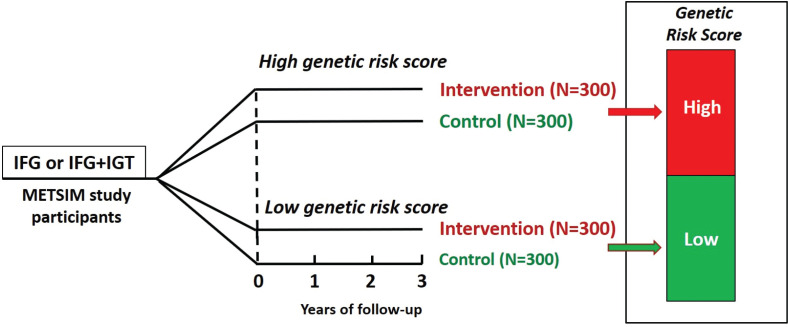
Study design of the T2D-GENE trial. Participants in the intervention and control groups of the trial are divided into two groups based on the genetic risk score for type 2 diabetes (low genetic risk and high genetic risk). IFG, impaired fasting glucose; IGT, impaired glucose tolerance. The duration of the intervention is follow-up time is 3 years for all participants.

The GRS in the present study was calculated on the basis of the number of risk alleles of 76 genetic variants increasing the risk for T2D as previously published (11). During the planning phase of the present study, less than 100 genetic variants increasing the risk for T2D had been published. A total of 600 participants were recruited and divided into two intervention groups (Fig. 1). Study participants, laboratory personnel, and clinical nutritionists were blinded with respect to the GRS of the participants throughout the study.

For both intervention groups (low GRS and high GRS), control groups of 300 participants (altogether 600 participants) of the METSIM study were selected and matched for GRS, fasting plasma glucose concentration, age, and BMI (Fig. 1). The rationale for these two control groups is to compare the incidence of T2D during the intervention between the two intervention groups and the two control groups.

### Calculation of the sample size

The average proportion of participants in whom IGT progressed to T2D in the Finnish DPS study was 3%/year in the intervention group and 6%/year in the control group (4). The absolute incidence of T2D was 32 cases/1,000 person-years in the intervention group and 78/1,000 person-years in the control group (4). Using the results from the Finnish DPS study and assuming similar rates of incident T2D (6%/year) in the control group, a total of 69 individuals of 300 (our sample size) or 23% will develop diabetes during a 3-year intervention. The corresponding numbers in the intervention groups are 31 of 300 or 10%, indicating a 13% difference in incident T2D between these two groups. If α = 0.05 and β = 0.95, only 60 individuals/group are needed to demonstrate statistically significant difference between the intervention and control groups. Our group-based intervention might be less efficient than individual-based approach in the Finnish DPS study. If the effect of intervention is 6% (about half of the effect of that of the Finnish DPS study), then 267 participants are needed. Assuming around 10% of dropouts in this trial, a sample size of 300/each group has a power of 95% to demonstrate statistically significant reduction in incident T2D in the intervention groups compared with the control groups.

## Methods

### Study visits

**Fig. 2:**
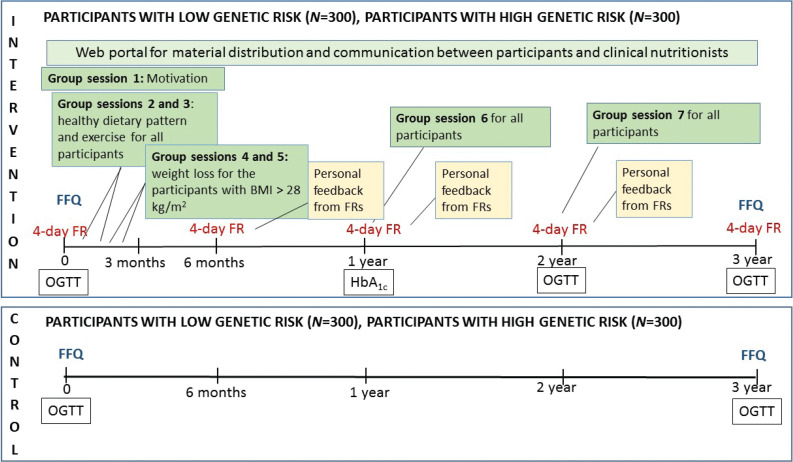
Intervention program. The participants belonging to the intervention group have seven group visits (group sessions on motivation; healthy dietary pattern and exercise; weight loss for those whose BMI was >28 kg/m^2^; and group sessions for all at 0, 1, 2, and 3 years for personal feedback from food records). Control groups have laboratory visits at 0 and 3 years, without any group sessions). FR, food record; FFQ, food frequency questionnaire; OGTT, an oral glucose tolerance test.

Participants in the intervention groups have four laboratory visits (0, 1, 2, and 3 years), and participants in the control groups have two laboratory visits (0 and 3 years) (Fig. 2). The study visits at 0 and 3 years include a questionnaire on physical activity at work and leisure time, smoking, alcohol use, history of diseases, and current medication, and dietary habits (food frequency questionnaire), blood pressure (three measurements 1 min apart), body weight, height, and bioimpedance for measure fat percentage. Laboratory measurements include an OGTT (plasma glucose and insulin concentrations at 0, 30, and 120 min), HbA1c, and concentrations of total LDL and HDL cholesterol, total triglycerides, alanine aminotransferase, creatinine, interleukin 1 receptor antagonist, and high sensitivity C-reactive protein. The three-factor eating questionnaire R18 (TFEQ-R18) (12) is used for the evaluation of participants’ eating behavior (cognitive restraint, uncontrolled eating, and emotional eating) during the intervention. In the intervention group, the 1-year visit includes measurement of non-fasting HbA1c. At the 2-year visit, the TFEQ-R18 questionnaire is filled, and body weight, blood pressure, and the laboratory measurements are performed similarly as at 0-year and 3-year visits.

### Intervention

The 3-year lifestyle intervention includes group sessions and the use of a website designed for this study, which enables the distribution of material and virtual discussions between the participants and clinical nutritionists. Printed material and discussions by phone are provided for those who do not have access to internet. The first year of the intervention includes 4–6 meetings depending on participant’s BMI (Fig. 2).

At the beginning of the intervention, a group session is organized (group session 1, about 150 participants at a time), where the aims of the study and the importance of lifestyle changes to reduce the risk of T2D are presented (Fig. 2). Group sessions 2 and 3 deal with healthy diet and physical activity. Participants needing more advice and support related to weight loss (BMI > 28 kg/m^2^) have two additional group sessions (4 and 5). About 5–40 participants/group have participated in group sessions 2–5. The spouses of the participants are invited to the group sessions lasting about 90 min. Group sessions are also organized at 1 and 2 years (group sessions 6 and 7).

Throughout the study, the participants in the intervention groups are contacted monthly by the nutritionists *via* the website to ensure participants’ activity for the intervention. Those participants not having internet access are contacted by mail. The participants are encouraged to contact the clinical nutritionists anytime they have questions or concerns.

### Diet

Dietary guidance in the intervention group is based on the Nordic (13) and Finnish nutrition recommendations, emphasizing appropriate energy intake, meal frequency, consumption of fruits, vegetables, and berries, quality of dietary fat, and carbohydrates, including sugar and fiber intakes (Table 1). Experience from the D2D project (14) has been utilized in the intervention as well. Overweight and obese participants are encouraged to lose weight. The minimum goal is not to gain weight during the intervention.

**Table 1:** Goals of nutrient composition of the intervention diet based on Nordic and Finnish nutrition recommendations

Nutrient	Goal
Vegetables, fruit, and berries (g/day)	≥500
Carbohydrate (E%, percent of energy intake)	45–60
Sucrose (E%)	<10
Fiber (g/day)	≥35
Fat (E%)	25–40
Saturated fat (E%)	<10
Monounsaturated and polyunsaturated fat (E%)	≥2/3 of total fat
Polyunsaturated fat (E%)	5–10
n-3 fatty acids (E%)	≥1
Monounsaturated fat (E%)	10–20

In addition to the quality and quantity of diet, aspects related to eating behavior are discussed in group sessions considering weight loss. The participants report their body weight weekly *via* the website or in writing (those who do not use internet). Dietary habits are evaluated by a food frequency questionnaire (FFQ) at baseline and at the end of the study. The FFQ is used previously in the FINRISK study (15), and with minor modifications and updates in the METSIM study (16). Four-day food records are used for measuring dietary intake enabling the evaluation of the achievement of the dietary goals. The intervention group keeps food records at baseline and at 6, 12, 24, and 36 months (Fig. 2). AivoDiet software (version 2.2.0.0, AivoFinland, Turku, Finland) is used for the calculation of dietary intake. Participants get personal written feedback on the food records based on the calculations to support their adherence to the dietary goals during the intervention.

### Physical activity

The goal regarding physical activity is brisk walking a minimum of 30 min per day at least 5 days a week. Walking could be replaced with other types of exercise (e.g. cycling, cross-country skiing, and housework such as leaves raking). Time used for physical exercise is recorded *via* website. Manual recording is possible for participants who do not have access to the internet.

### Laboratory analyses

Laboratory analyses are performed in the laboratory of Internal Medicine at the University of Eastern Finland. Insulin sensitivity is calculated as Matsuda index of insulin sensitivity and insulin secretion as Disposition index, as previously described (11).

### Statistical analyses

The primary analyses for the outcomes (incident T2D) in the time-to-event analyses will be based on a Cox proportional-hazards model with the study group (intervention and control) as a covariate in the low and high GRS groups. Linear regression will be used to evaluate the effect of intervention on insulin sensitivity, insulin secretion, the glucose AUC, and other continuous variables. Comparison between the study groups at baseline and at the end of 3-year intervention will be carried out with t-test (continuous variables) intervention and chi-square test (non-continuous variables).

### Ethical issues

The study plan has been approved by the Ethical committee of the Hospital District of Northern Savo (no. 71/2016). This study is conducted in accordance with the ethical standards laid down in the 1964 Declaration of Helsinki and its later amendments. The participants received both oral and written information about the study, and they gave a written informed consent. They have right to discontinue the study without telling the reason.

### Time table

The first participants started the intervention in April 2016, and the recruitment was completed in April 2018. Altogether 635 participants were recruited. The first participants finished the 3-year intervention in April 2019, and the last participants will finish it in September 2021. The last participants in the control group will have a 3-year visit in September 2021.

### Baseline data

The baseline characteristics of the participants in the intervention groups are presented in Table 2 (low and high genetic risk groups combined). The mean age of the participants was 65.0±5.9 years (mean ± SD) at the baseline, and they were obese with the mean waist circumference higher than recommended. Fasting plasma glucose concentration was within the range of IFG (5.6–6.9 mmol/l), 2-h glucose concentration <11.1 mmol/l, and HbA1c (<48 mmol/l or <6.5 %). Blood pressure and concentrations of total and LDL cholesterol were slightly higher than recommended.

**Table 2:** Baseline characteristics of the participants in the intervention group (*n* = 635)

Variables	Range	Mean ± SD
Age, years	51–75	65.0±5.9
Body mass index, kg/m^2^	25.0–48.5	28.7±3.2
Waist circumference, cm	84.0–149.0	103.3±9.0
Systolic blood pressure, mmHg	101–193	134±15
Diastolic blood pressure, mmHg	60–117	84±9
Fasting glucose, mmol/l	5.6–6.9	6.0±0.3
2-h glucose, mmol/l	2.7–10.9	6.3±1.6
HbA1c, mmol/mol	29–45	37.5±3.1
Total cholesterol, mmol/l	2.49–7.91	5.03±1.00
Low density lipoprotein-cholesterol, mmol/l	1.05–5.88	3.10±0.90
high density lipoprotein-cholesterol, mmol/l	0.60–2.80	1.38±0.36
Total triglycerides, mmol/l	0.46–5.73	1.37±0.67

## Discussion

Our study will be the first study to compare the effect of lifestyle intervention on the prevention of incident T2D and the worsening of hyperglycemia in people with high genetic risk of T2D compared with people with low genetic risk of T2D.

If the effects of the lifestyle intervention are independent from the genetic risk of the participants, the implications of our study will be of great importance for the entire T2D research community, health care providers, and individuals at high risk for T2D. In this case, lifestyle intervention is beneficial for all individuals at risk for developing T2D, independently of genetic risk. If the effects of lifestyle intervention depend on the genetic risk, then individualized intervention programs are needed based on the GRS in people at high risk for T2D.

Due to the high number of people at risk for T2D in most countries, it will be challenging to organize individualized lifestyle counseling. In our study, lifestyle counseling is group-based, and a special website designed for this study is used for nutritionists-participant and participant-nutritionists contacts. From the resources point of view, it is important to examine whether a group-based intervention is effective similarly to a personal lifestyle counselling, and this study is likely to give an answer to this important question.

There are several previous lifestyle intervention studies, in counseling which the efficacy of lifestyle intervention on the prevention of T2D has been reported. Most of these studies have been performed in participants with IGT. In the present study, participants with IFG with or without IGT have been included. Most of the studies have combined diet and physical exercise, and a minority of the studies have only dietary intervention (17). In the systematic review and meta-analysis, the risk ratio (RR) was 0.68 (95% CI 0.5; 0.84) for dietary interventions and 0.59 (0.51; 0.69) for interventions combining diet and physical activity (17). In a more recent SRMA, the RR was 0.53 (0.41; 0.67) for interventions combining diet and physical activity (18).

In previous studies, the interventions have mainly been individual based. In the Finnish DPS, the participants met clinical nutritionist seven times during the first year of the intervention and three times per year thereafter (4). In the DPP, the participants were contacted by phone (5). In real-world setting, a group-based approach has been studied. In an SRMA of real-world lifestyle modification aiming for T2D prevention, participants receiving group counseling by health care professionals had an RR of 0.67 (0.49; 0.92) as compared with control participants (19).

The most significant challenge of our study is that the group-based intervention utilizing a specific website is not efficient enough. However, due to an increasing number of people with impaired glucose metabolism or T2D, it is important to test this type of intervention since individual counseling is usually not possible, not at the present time and even less in the future, since the number of people at risk for T2D is increasing.

The results of the present study will reveal whether a lifestyle intervention (diet, physical activity, and weight maintenance/weight loss) is as effective in the prevention of incident T2D and the worsening of hyperglycemia in people with high number of T2D risk alleles compared with people with low number of T2D risk alleles, and whether a group-based intervention utilizing a website designed for this purpose is as effective as an individual-based intervention.

## Data Availability

Not applicable at this point (protocol article).

## Additional declaration

This study was conducted in accordance with the ethical standards laid down in the 1964 Declaration of Helsinki and its later amendments.
